# Building community trust through medical education: a four-year descriptive analysis of a national grant program

**DOI:** 10.3389/fpubh.2026.1862984

**Published:** 2026-07-06

**Authors:** Mary S. Hedges, Randl Dent, Timothy Lynch, Maria de Miguel

**Affiliations:** 1 Department of Medicine, Division of Community Internal Medicine, Mayo Clinic, Jacksonville, FL, United States; 2 ABIM Foundation, Philadelphia, PA, United States; 3 Department of Medicine, Division of General Medicine, Columbia University Medical Center, New York, NY, United States

**Keywords:** community, grant funding, innovation, interprofessional, medical education, trust

## Abstract

Community engagement and trust are vital to medical education and improving health. The experience of national level, extramural seed grant programs to support faculty in both education and health care delivery with the focus of building trust has not been previously reported. The authors reviewed a total of four cohorts of the “Building Trust: Advancing Health Equity” grant recipients supported by the ABIM Foundation and other funding partners. Over $1.5 million was awarded from 2021 to 2024, which represented 92 grants ranging from $2,500–$40,000 each. The geographic regions receiving the most grants and funding were the middle and south Atlantic, and the Pacific regions in the United States. University programs received more grants and funding than affiliated or community programs. The majority of project types primarily focused on developing educational curricula for internal medicine residents (68%) and engaging with local communities (19%). The approach to implementation often included service-learning and community experts (29%), quality improvement and patient care delivery methods (22%), and traditional curriculum development (21%). The most common education topic themes were health equity and social determinants of health (43%) and addressing bias in care delivery and learning environment (20%). The Building Trust grant program reflects the unique needs of local communities and supported innovative practices and educational techniques. Implementation support was provided to grantees through shared experiences and mentorship. Evaluating outcomes upon completion of grants will be an imperative next step. This serves as a model that can inform institutions and other professional organizations approaching implementation of seed grant funding.

## Introduction

Community trust and engagement with health systems and medical education is vital to improve health for all groups (1). Training on the social and structural determinants of health is well established as a core value of medical education at the undergraduate and graduate levels (2, 3). Furthermore, experiential trainee learning opportunities that engage the local community have been shown to have significant transformative impact for students (4, 5). However, faculty face significant limitations in time, financial and administrative support, academic recognition, and academic mentoring to create innovative educational models or health care delivery improvements to promote health equity in their community (6, 7). Projects that combine disciplines or partner with community members outside the clinical space can present a unique challenge in securing institutional funding and support.

Grant programs can support faculty in piloting new programs in education, workforce development, and healthcare delivery that generate creative solutions to complex health problems. Medical education research funding is critical for high impact research but can be difficult to obtain, especially for clinician-educators (8). Large, extramural research grants often require research teams and may carry a high administrative burden. Faculty on the front lines of teaching and caring for patients have the expertise to create innovative community-oriented programs, but often not the research apparatus to support applying for large scale grants.

Intramural grant programs can support site specific innovative projects developed by faculty to promote health in local communities and encourage healthcare system improvement (8–11). Large scale, extramural seed grant programs have shown value in generating innovation in UME teaching methods and GME training programs (12). The first four cohorts of the “Building Trust: Advancing Health Equity” grant program were supported by several national professional medical organizations. The program solicited both education-driven proposals and proposals that focused on improving healthcare delivery to promote trust, health equity, and address the unique needs of the local community (13). At various times, the Alliance for Academic Internal Medicine (AAIM) and the American College of Physicians (ACP) co-sponsored the program with the ABIM Foundation. The Gordon and Betty Moore Foundation and the Josiah Macy Jr. Foundation have also provided financial support for the program. Recipients of grant awards received additional support from the ABIM Foundation, including engagement in a grantee learning network, coaching support, connections to other grantees, and additional financial support and encouragement to present or publish scholarly outcomes from their grant work.

The program was conceived in 2020, at a time of historic distrust in the medical system and increasing social awareness of health disparities and inequities (14). The program also coincided with an update of the Accreditation Council for Graduate Medical Education (ACGME) milestones for Internal Medicine residencies to include skills in understanding and addressing the social determinants of health (15, 16). Using Mayer et al.’s (17) integrative model of organizational trust, trust is defined as “a willingness to be vulnerable, grounded in the expectation that others will act with competence, integrity and benevolence”. Trust is the foundation of an effective healthcare system, including at the individual level, the team level, and at the system level. Studies have shown a positive correlation between enhanced trust in healthcare professionals and more favorable health outcomes for patients (18).

With the grant program’s goal of building trust with communities, potential projects were evaluated based on their innovation, feasibility and sustainability, their intentional focus on building trust and health equity, their engagement of multiple health professions, and their partnerships with their local communities. The aim herein is to provide a descriptive thematic analysis of the grants awarded and the approaches taken by the first four cohorts of the Building Trust grant recipients, in order to explore and characterize the local training needs related to trust and equity in the healthcare system that were addressed by grants.

## Grant review

A total of four cohorts of Building Trust grant recipients were reviewed by the authors of this article, which included 92 grants that distributed $1,577,500, within the date ranges 2021–2024 (19–22). Grant proposal information was provided by the ABIM Foundation for the authors to review, with full proposal descriptions available for 91 of the 92 grants. Subject areas analyzed included total dollar amount by academic year, geographic region, institutional setting, grant recipient academic position, as well as grant project type, approach, and topic theme.

Project type, approach, and topic theme were defined using an inductive qualitative content analysis approach. Two authors from the AAIM innovation grants committee served as independent reviewers for the coding process, reviewing each proposal to code the main purpose of the project, and if applicable, a secondary project type was additionally coded. Through iterative review, the two coders organized the codes into broader themes and subsequently assigned two sub-themes for each project—the approach, which is the primary method used to accomplish the grant objectives, and the topic theme, which reflects the main area of focus for the project. For the purposes of analysis, the approach and topic theme coded were the ones that were most predominant in the proposal, as many projects used multiple approaches and covered more than one theme in the grant project. A third author, from the ABIM Foundation, provided oversight and reviewed final categorical themes for consistency and confidence in the coding process (Table 1).

**Table 1:** Categories of grant project type, approach, and topic themes.

Project types	*N* (%)
Education and training	62 (68%)
Community engagement	17 (19%)
Pathway programs	4 (4%)
Faculty development	3 (3%)
Workforce development	3 (3%)
Interprofessional skill development	2 (2%)
Approaches	
Service-learning, immersion, community expert as teacher	26 (29%)
Quality improvement and patient care delivery improvements	20 (22%)
Curriculum development	19 (21%)
Simulation experiences	10 (11%)
Faculty support	7 (8%)
Mentorship program	5 (5%)
Community healthcare program and health education	4 (4%)
Topic themes	
Health equity and social determinants of health	40 (43%)
Bias in care delivery and learning environment	18 (20%)
Community health	10 (11%)
Advocacy	8 (9%)
Communication skills and health literacy	4 (4%)
Homelessness and food insecurity	4 (4%)
Academic medicine faculty and health professions training	3 (3%)
Hospital-based/POCUS	3 (3%)
Home visits	1 (1%)

*N* = Number of projects (% = percentage of total projects).

### Grant funding by academic year

Grants ranged from $2,500–$20,000 per project in the first year, then $10,000–$40,000 per project in subsequent years. Overall, there was a trend towards fewer total grants for higher dollar amounts for each successive year. The initial grant year 2021 included 32 grant recipients that received a total amount of $287,500. In 2022, 2023, and 2024, there were fewer grant recipients (24; 20; and 16 respectively) for higher total dollar amounts ($400,000; $470,000; and $420,000 respectively).

### Geographic settings of grant recipients

Geographic region was defined using the Association of American Medical Colleges (AAMC) geographic regions (Supplementary material S1) (23). The AAMC geographic regions with the most grants and funding awarded were the Middle Atlantic (20/91 grants, 22% of grants; $267,000 funding), the Pacific (17/91 grants, 19% of grants; $285,000 funding), and South Atlantic (15/91 grants, 16% of grants; $287,500 funding). The AAMC geographic region with the fewest grants and least funding was East South Central (2/91 grants, 2% of grants; $30,000 funding).

**Figure 1:**
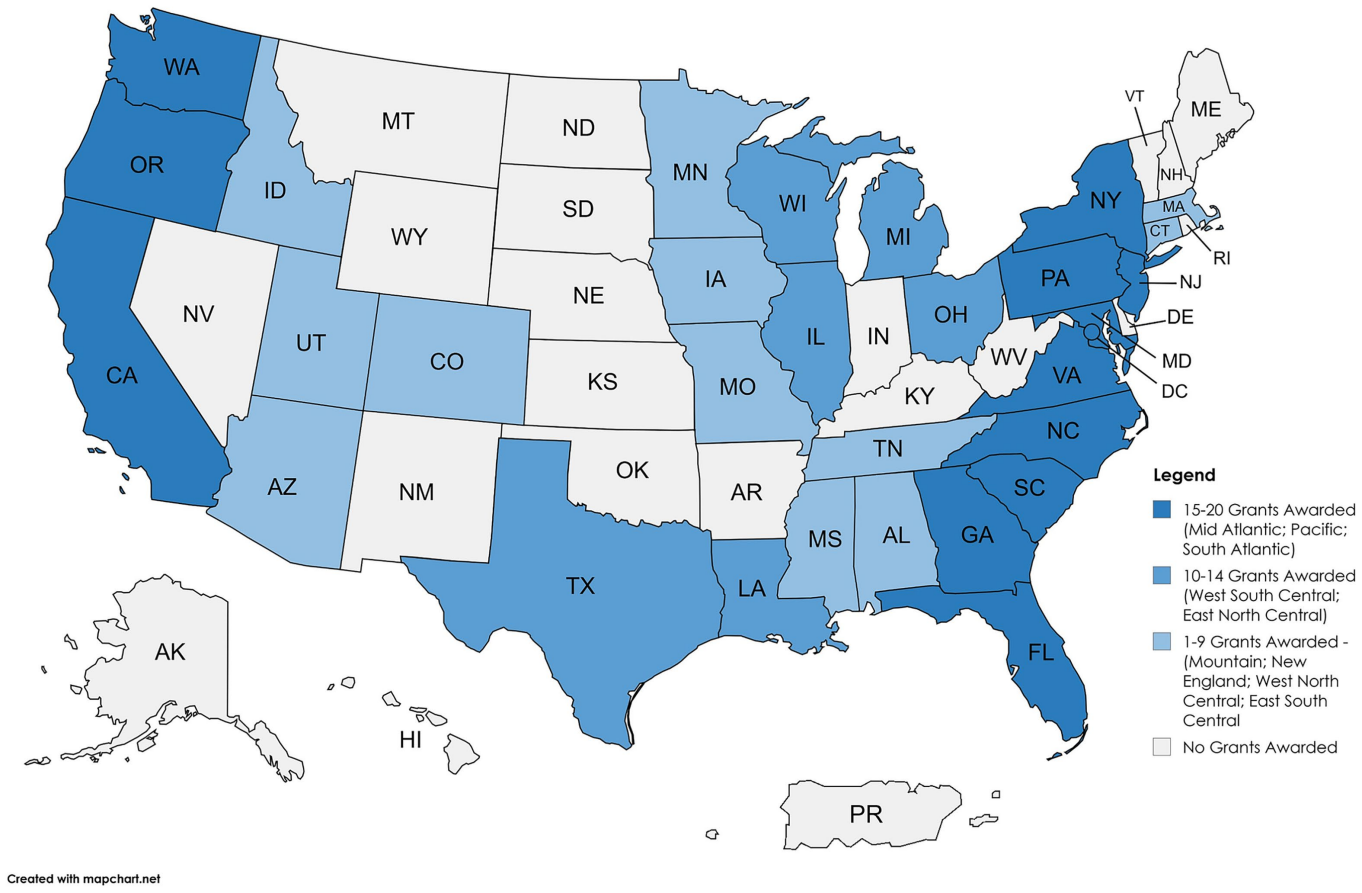
Distribution of the building trust grants across the U.S. by AAMC region from 2021 to 2024. AAMC Regions with higher numbers of grants awarded (15–20) – Dark Blue Middle Atlantic (20), Pacific (17), South Atlantic (15). AAMC Regions with medium numbers of grants awarded (10–14) – Medium Blue West South Central (12), East North Central (11). AAMC Regions with lower numbers of grants awarded (1–9) – Light Blue Mountain (6), New England (5), West North Central (5), East South Central (2). States from any region that received zero grants – Gray.

Distribution patterns were noted aside from AAMC geographic regions. Specifically, there were no grant recipients outside of the continental United States (Hawaii, Alaska, Puerto Rico). The central Plains states were noted to have received very few grants (Figure 1).

### Institutional settings of grant recipients

Institutional setting was defined using the self-reported descriptor from the Residency Explorer website, which included: University Hospital, Affiliated Hospital, Community Hospital, or Other (24, 25). University programs received the highest total numbers of grants and most total funding (63/91 grants, 69% of grants; $1,052,500 funding), followed by Affiliated programs (20/91 grants 22% of grants; $350,000 funding), Community programs (7/91 grants, 8% of grants; $115,000 funding), then Other (2/91 grants, 2% of grants; $60,000 funding).

### Academic position of grant recipients

There was a wide range in the seniority and academic rank of the grant recipients, ranging from medical students, residents, and chief residents to senior faculty, including deans, associate deans, program directors, associate program directors, and clerkship directors. The academic rank of the grant recipients ranged from instructor in medicine to professor of medicine, and most commonly was assistant or associate professor academic rank.

### Primary and secondary project types of grants

The majority of grants focused on the development of educational programs or curricula for internal medicine residents (62/91 grants, 68% of grants). Several other projects prioritized community engagement by involving community members in the design or implementation of educational activities in their grant project (17/91 grants, 19% of grants). The most common secondary project types were community engagement (28/91 grants, 31% of grants) and interprofessional collaboration (23/91 grants, 25% of grants).

### Approaches of grants

Many projects utilized service-learning teaching methods, which include activities in which residents were immersed in the local community and engaged community experts and leaders as educators (26/91 grants, 29% of grants). Many projects focused on utilizing quality improvement methods to improve patient care delivery (20/91 grants, 22% of grants). Several projects utilized traditional didactic methods, including lectures, seminars, grand rounds, and lunch and learns (19/91 grants, 21% of grants). Other less frequent strategies worth noting included simulation-based education, community focused events, development of pathway programs to support underrepresented trainees, and faculty development efforts.

### Topic themes of grants

The most prevalent education topic was a high-level education on social determinants of health and impacts on health equity for the local communities (40/91 grants, 44% of grants). These projects aimed to integrate structural and social influence on health into clinical training for resident physicians. A significant number of grant projects explored the learning environment including the impact of implicit bias in clinical care, with a focus on developing strategies to recognize bias and mitigate its impact (18/91 grants, 20% of grants). Several initiatives also focused on educating community members on health topics relevant to their lived experience and needs (10/91 grants, 11% of grants). Most projects educated residents on multiple topics related to social determinants of health and community resources. Several projects also used multiple approaches, for example combining traditional didactic methods with experiential learning based in their local community (Table 2).

**Table 2:** Examples of topic themes of grants awarded.

Topic theme	Grantee examples
Health equity/social determinants of health	Novel curriculum for residents that focused on interpersonal and communication skills training for diverse patient populations. *Approach: curriculum development* Teaching students to use a dashboard to identify health disparities and collaborate with multidisciplinary teams to develop and implement quality improvement projects that promote health equity *Approach: quality improvement*
Bias in care delivery and learning environment	Algorithm to identify stigmatizing language, curriculum for residents to modify use, and tool created to review stigmatizing language. *Approaches: quality improvement & curriculum development* Workshop to understand how to identify and reduce race-based diagnostic errors (e.g., eGFR for kidney function). *Approach: curriculum development*
Community health	Promote health and increased patient education in predominantly Hispanic populations served by a student-run clinic, inviting residents to educate patients about managing diabetes and hypertension and colorectal cancer screenings. *Approaches: service learning & health education*
Homelessness and food insecurity	Established consistent, quality healthcare for the unhoused community by meeting patients geographically where they are, inspired by the street medicine model of care. Created a medical home that provided both urgent and primary care services. *Approaches: service learning & community health program* Implemented an interdisciplinary food prescription program in a residency training with recurrent patient interaction and monitoring patient outcomes. *Approach: community health program*

### Key insights

Several common themes were identified throughout the extensive review of the multiple grants awarded. Clearly defined grant criteria helped ensure alignment with the mission of the program, specifically a community engagement component, an interdisciplinary or interprofessional component, and a focus on building trust. The grant encouraged an innovative mindset, creativity, and flexibility for the projects to respond to unique and specific local community needs. While many funded projects were new initiatives, this was not a requirement; and some grants supported existing initiatives that needed additional support to continue or to be expanded.

This grant program attracted faculty from a broad variety of geographic distribution and academic experiences. Each grant tailored its approach to the needs of the community it served, an important aspect of building trust. The vast majority of grants sought to implement their initiatives through the development of curricula for internal medicine residents and implementing community-based health programs, highlighting the ongoing need for support to integrate these topics into traditional medical training. These models could be adapted and tested in other community settings, tailored for local community needs.

The topics covered and approaches utilized varied greatly, with grants aiming to educate residents on multiple related topics and using a variety of methods to accomplish their grant objectives (e.g., community health programs and curriculum development). While the main topics that emerged are universally relevant, faculty utilized their expertise from patient care and teaching with local resources and community experts to meet the unique needs in their community. Efficient reporting of data to track outcomes is important, minimizing paperwork requirements while clearly outlining important outcomes to be tracked, as the grants themselves provided limited salary support for faculty.

Sharing the experiences of each grantee, both successes and failures, can be helpful to others seeking to implement similar ideas. To support this, the ABIM Foundation continues to cultivate a learning community for grantees that includes an online resource hub and grantee contact directory. Additionally, to allow grantees to effectively adapt their projects as needed to meet the needs of the community, grant program staff were amenable to grant modifications. This allowed flexibility to pivot and adjust projects as roadblocks arose.

Professional organizations committed to advancing the core values of medicine can leverage grant programs to generate creative, practical approaches to address critical topics and local community needs. This grant program sought to address a persistent unmet need by supporting faculty in implementing interventions that center on building trust and partnering with community organizations with the ultimate goal of improving community health. Clinician-educator faculty can feasibly apply for and complete small grants to support work on topics that are traditionally not well supported by academic institutions, such as community outreach and inter-professional work.

The range of faculty and academic sites shows the depth and breadth of the need for faculty support working on these important health equity topics. Furthermore, the number of programs that utilized these grants to work more directly with the community suggests the lack of traditionally available funding for work outside of the clinical setting that is critical to building community trust. Future considerations could include targeted dissemination of the Request for Proposals to geographic areas where fewer grants were received over the last four years. Community-based programs were also less likely to receive grants, and thus a grant program dedicated to community-based training programs could help bridge this gap. Institutions are additionally encouraged to prioritize and value work that has societal impact and improves public trust when considering academic or leadership promotion (26). This grant program provided extramural grant support and recognition of community-focused faculty, a critical step for academic faculty promotion and elevation of this topic within academic institutions.

## Limitations

The current study is descriptive in nature, and the qualitative analysis serves to highlight the topics and approaches supported, and does not include projects that were not chosen but may also reflect important community needs. As the grant projects are currently underway, it was not possible to analyze completed project outcomes. Upon completion of grant projects, it will be imperative to assess the grant outcomes across the cohorts. Many indicators for success should be considered, including post-intervention outcomes, demonstration of sustainability and scalability, long-term effectiveness, applying for and obtaining subsequent funding, sharing of knowledge through presentations or publications, and numbers of trainees or patients impacted through this work. Lastly, there is the potential for positivity bias as the authors are affiliated with organizations supporting the grants program. This was mitigated through implementing an exploratory and descriptive analysis without a hypothesis to be tested.

## Conclusion

This study provides a descriptive analysis of a national medical education grant program focused on community engagement, health equity, and trust. Over four years, funded projects most commonly emphasized curriculum development, service-learning approaches, and interprofessional collaboration, with notable variation in inclusion by geographic region and institutional setting. Future evaluation of outcomes of each grant will be strategically important for sharing best practices and identifying gaps. This serves as a model that can inform institutions and other professional organizations approaching implementation of seed grant funding.

## Data Availability

The original contributions presented in the study are included in the article/Supplementary material, further inquiries can be directed to the corresponding author.
