# Supplementary material for: Building community trust through medical education: a four-year descriptive analysis of a national grant program
Source: Front Public Health. 2026 Jul 6;14:1862984. doi: 10.3389/fpubh.2026.1862984 (PMC13381442; doi:10.3389/fpubh.2026.1862984)
Supplement: Supplementary file 1 [file Table_1.docx]

**Supplementary Material S1**

Geographic regions as listed by AAMC

- New England: Connecticut, Maine, Massachusetts, New Hampshire, Rhode Island, Vermont.
- Middle Atlantic: New Jersey, New York, Pennsylvania.
- East North Central: Illinois, Indiana, Michigan, Ohio, Wisconsin.
- West North Central: Iowa, Kansas, Minnesota, Missouri, Nebraska, North Dakota, South Dakota.
- South Atlantic: Delaware, District of Columbia, Florida, Georgia, Maryland, North Carolina, Puerto Rico, South Carolina, Virginia, West Virginia.
- East South Central: Alabama, Kentucky, Mississippi, Tennessee.
- West South Central: Arkansas, Louisiana, Oklahoma, Texas.
- Mountain: Arizona, Colorado, Idaho, Montana, Nevada, New Mexico, Utah, Wyoming.
- Pacific: Alaska, California, Hawaii, Oregon, Washington.
